# Differences in New Zealand Hop Cultivars Based on Their Unique Volatile Compounds: An Integrated Fingerprinting and Chemometrics Approach

**DOI:** 10.3390/foods10020414

**Published:** 2021-02-13

**Authors:** Victoria Purdy, Biniam Kebede, Ron Beatson, Kerry Templeton, Patrick Silcock, Graham T. Eyres

**Affiliations:** 1 Department of Food Science, University of Otago, P.O. Box 56, Dunedin 9054, New Zealand; victoria.purdy@postgrad.otago.ac.nz (V.P.); biniam.kebede@otago.ac.nz (B.K.); pat.silcock@otago.ac.nz (P.S.); 2 The New Zealand Institute for Plant and Food Research Limited, 55 Old Mill Road, RD3, Motueka 7198, New Zealand; Ron.Beatson@plantandfood.co.nz (R.B.); Kerry.Templeton@plantandfood.co.nz (K.T.)

**Keywords:** *Humulus lupulus*, New Zealand hops, volatile compounds, HS-SPME-GC-MS, fingerprinting, chemometrics

## Abstract

Hop aroma characteristics originate from hop essential oils, which have complex chemical profiles that remain poorly understood, particularly for New Zealand hops. The aim of this study was to determine volatile compounds that distinguish New Zealand hop cultivars. Untargeted fingerprinting methods based on headspace gas chromatography mass spectrometry (GC-MS) were used to analyse nine hop cultivars. A total of 61 volatile compounds were identified as compounds that differentiated the commercial hop varieties using advanced chemometrics and feature selection techniques. Similarities in volatile composition were found between Wakatu, Wai-iti™ and Kohatu^®^, which are rich in alcohols. Another grouping was found between Waimea™ and Nelson Sauvin™, where ketones and esters were commonly found. Rakau™ was distinct from the other eight cultivars, distinguished by 2-methylbutyl 3-methylbutanoate and methanethiol hexanoate. Riwaka™ contained the greatest number of discriminating volatile compounds when compared to other cultivars, which was dominated by terpenoids, such as geranyl 2-methylbutanoate, perillene and D-limonene. The chemical fingerprinting approach successfully identified volatile compounds that had not been previously found in New Zealand hop cultivars and that discriminated the commercial cultivars. The data obtained in the present study further extend the knowledge of New Zealand hops and will help facilitate targeted breeding.

## 1. Introduction

Hops (*Humulus lupulus*) are grown in moderate climates all around the world including Europe, North America, Japan, Australia and New Zealand [1]. Hop plants belong to the Canabinaceae family, which consist of *Humulus* and *Cannabis* genera. The *Humulus* genera consists of two species, *Humulus lupulus L.* and *Humulus japonicas* [1]. *Humulus lupulus L.* is what is commonly referred to as the hop plant, where *Humulus japonicas* is only produced as an ornamental plant.

Hops, one of the four primary ingredients used to make beer, contribute bitterness and aroma characteristics to beer. It is known that α-acids from hops are responsible for beer’s bitterness and essential oils are responsible for the aroma characteristics [2]. Some of the common odour-active volatile compounds in hops include terpenes, esters, ketones and aldehydes [3]. Nevertheless, due to the complexity and quantity of aroma compounds, it has been difficult in the past to determine which compounds are unique to different hop cultivars [4]. With the recent increasing trend in craft beers, and drive for beers with unique flavours, it is important to increase our understanding of the aroma compounds in hops that contribute to these distinct flavour characters.

Terpenes are organic compounds found in plants that are made up of a number of 5 carbon 2-methylbutane (isoprene) units [5]. Terpene derivatives can also be present with a range of different functional groups, which change the odour character and activity [2,4]. Volatile esters are important flavour-active compounds in hops where certain cultivars are classified according to their fruity characteristics [3]. Ketones have been described in literature to have citrus and fruity notes [3].

The process of breeding hops is complex and time-consuming, and can take up to 20 years [6]. As the trend for craft beer increases, the demand for distinctly flavoured hops has also increased. New Zealand has bred a number of distinctly flavoured hop cultivars and currently aims to increase this number by breeding more cultivars that are unlike those currently on the market [6]. Since the 1940s, when black root rot affected hops, New Zealand has focused on a breeding programme that focused on breeding for disease resistant and seedless hops. This led to New Zealand being the first country to commercially produce seedless triploid cultivars [7]. Brewers prefer to use seedless hops as hops with seeds can have negative effects on the brewing process [7]. The New Zealand breeding programme begins with the parent hop plants being crossed to create seedlings [6,8]. Selections are then made and measurements are taken, including chemical analysis measuring the α and β acids and essential oils, sensory analysis and pilot brewing trials [8]. If the hop plants are successful in these analyses, they will move onto grower trials, where they are grown in different areas.

Brewing trials are also conducted to see how the hops perform in beer [8]. After these stages successful hop cultivars will be commercialised and become available for use by brewers. The New Zealand hop breeding programme has been very successful in creating hops with distinct aroma characteristics. Although New Zealand has many distinct hop cultivars, their chemical profiles are not well characterised. Although the profile of Nelson Sauvin™ has been published [9,10], the majority of New Zealand hop cultivars are at best poorly characterised. In this context, state-the-art chemical fingerprinting methods have a huge potential to add to knowledge on the characteristic volatile compounds present in these hops.

Fingerprinting is an untargeted analytical approach aiming to detect as many chemical compounds as possible present in the particular food matrix [11,12]. Chemical fingerprinting considers all compounds detected in the investigated food fraction, which will improve the chance of novel discoveries in exploratory research [13]. Fingerprinting approaches consist of several steps, in which the main ones are sample preparation, separation, detection and data analysis. It is important that along each of these steps volatile compounds are not lost. Headspace methods ensure volatiles are not lost or degraded during sample preparation as they are in a closed system [14]. Hence, in the present work, a headspace solid phase micro-extraction gas chromatography mass spectrometry (HS-SPME-GC-MS) fingerprinting method was implemented to analyse the volatile fraction of hop cultivars. As chemical fingerprinting generates a large data set, advanced chemometrics and feature selection methods are used to classify the samples, determine patterns and trends and identify discriminant volatile compounds. By discovering which volatile aroma compounds discriminate the different hop cultivars, hop breeders can use this information and methods to help develop new unique cultivars. This will facilitate expansion of the hop industry, reduce breeding time and costs and add value to the increasing craft beer industry.

Fingerprinting has been previously conducted to link chemical attributes to genetics in hops grown in Australia [15]. This study was able to identify differences in chemical compounds between the different hop cultivars using principal component analysis (PCA). The present study differs from this study as further data analysis was conducted to identify the specific compounds that are most different between hop cultivars by using partial least squares regression and feature selection techniques. Fingerprinting has not yet been conducted on New Zealand hops using such in depth data analysis, thus allowing the New Zealand hop industry to identify the compounds that characterise their hops and extend their knowledge for future hop breeding by providing chemical phenotype targets.

The objective of this research was to determine which volatile compounds are characteristic for different New Zealand hop cultivars using a chemical fingerprinting and chemometrics approach to help facilitate the hop breeding programme in New Zealand.

## 2. Materials and Methods

### 2.1. Materials

Nine different commercially available New Zealand hop cultivars from the 2017 harvest (Nelson Sauvin™, Riwaka™, Motueka™, Wai-iti™, Waimea™, Kohatu^®^, Rakau™, Wakatu and Taiheke^®^) were analysed. These cultivars were later compared to nine advanced selection cultivars (hops that are in the New Zealand breeding programme).

Hop characteristics can be altered by a number of factors including their environment, harvest time and the way in which they are handled. To decrease the amount of variation between samples, all hop samples were compressed, dried flower cones (kiln dried at 62–65 °C for 8 h to a target moisture content of 8%) grown in the same hop field at The New Zealand Institute for Plant and Food Research Limited, Motueka, Nelson (41°5′46.166″ S, 172°58′27.021″ E). Samples were vacuum packed and couriered to the University of Otago where they were stored (−20 °C) prior to analysis.

### 2.2. Sample Analysis

To mimic the brewing process, obtain a representative sample and ensure the SPME fibre was not overloaded with volatile compounds, a hot water extract was prepared based on literature [16,17] with slight modification. Dried hop cones were individually ground (15 g) using liquid nitrogen (BOC gases, Auckland, NZ, USA) and a mortar and pestle. Subsamples (1.25 g) of each ground hop powder were placed into three separate 250 mL bottles (Schott Duran^®^, Mainz, Germany) that contained 250 mL milli Q water at 90 °C. Each bottle containing the hop sample was placed in a 90 °C hot water bath for five minutes before transferring the bottles into an ice water bath for one hour to cool, resulting in three hop extracts for each of the different hop cultivars.

Each hop extract was run in duplicate to give six analytical measurements for each hop cultivar. Hop extract (8 mL) was placed into a labelled vial containing the 2.5 g NaCl (BDH, Poole, UK) and immediately sealed with a PTFE-lined lid [18]. Each vial was mixed using a vortex mixer (Chiltern MT 17) for 30 s and stored frozen (−20 °C) until analysis. Water blank vials were prepared by pipetting milli Q water (8 mL) into 13 × 20 mL headspace vials containing 2.5 g analytical grade NaCl. Vials were stored frozen until analysis using gas chromatography mass spectrometry (GC-MS).

Once defrosted, prepared vials were randomly ordered and placed in a holding tray of the GC-MS instrument (Agilent Technologies 6890N with Agilent PAL3 RSI 85 Autosampler; Palo Alto, CA, USA). Water blank vials were run every 10 GC-MS analyses to monitor any carryover. Each sample vial was incubated at 40 °C for 5 min with the agitator turned on. The SPME fibre (divinylbenzene/carboxen/polydimethylsiloxane coated) was exposed to the vial headspace for 30 min at 40 °C for extraction [18].

Extracted volatiles were desorbed in the GC inlet at 230 °C for 5 min (2 min splitless at 1 mL·min^−1^; followed by purge of 50 mL·min^−1^) with helium as the carrier gas. Separations were conducted with a polar ZB Wax GC column (Phenomenex, Torrance, CA, USA; 60 m, 320 μm internal diameter, 0.5 μm film thickness). The carrier gas was helium using constant flow mode (1 mL·min^−1^ flow rate). The oven program was set to have an initial temperature of 50 °C with a hold of 5 min followed by an increase to 210 °C at 5 °C·min^−1^, then increasing to a final temperature of 240 °C at 10 °C·min^−1^. The mass spectrometer (Agilent Technologies 5975B VL MSD with triple axis detector) was set up with a mass spectrum scanning range of 30–300 *m/z.* The MS ion source and MS quadrupole temperature was set at 230 °C and 150 °C, respectively.

### 2.3. Data Preprocessing and Multivariate Data Analysis (MVDA)

An automated mass spectral deconvolution and identification system (AMDIS, National Institute of Standards and Technology (NIST), Version 2.72, 2014) was used to deconvolute co-eluting peaks on the total ion chromatogram obtained with the HS-SPME-GC-MS analysis. The deconvoluted spectrums obtained after AMDIS were analysed using mass profiler professional (MPP) (Agilent Technologies, Version 14.9, 2017) to align the peaks and filter and remove irregular and non-reproducible peaks. MPP produced a final data table, which was used in the following multivariate data analysis techniques [11].

The multivariate data analysis (MVDA) was performed using Solo (Matlab Version 8.3.0.532, 2018, Eigenvector Research, Wenatchee, WA, USA). MVDA tools enable extraction of relevant information out of the large data sets by reducing dimensionality of the data and studying the correlation patterns. These multivariate techniques transform the large number of original variables into just a few, manageable variables that can maximally explain the variation in the data so that analytical information of importance is emphasized.

Principal component analysis (PCA) was run as an exploratory/unsupervised learning tool to describe the data as no additional knowledge (e.g., Y variable) besides the raw data (X variable) is required to describe the data set. This enabled the detection of outliers and common groupings between samples and evaluation of the relationships between samples and variables.

Partial least squares discriminant analysis (PLS-DA) was used to maximally describe the separation between the sample classes in the multivariate space according to their class membership. PLS-DA includes the available knowledge on a dependent response Y variable to obtain a latent variable model that optimally describes the response variable. Hence, PLS-DA is a supervised statistical technique. Results from the PLS-DA gave visual representations via score and loadings plots for the interpretation of results. These were combined into bi-plots, which graphically illustrated how samples relate to each other (scores) and the importance of each variable to the separation (difference) between classes (loadings). To select the optimal number of latent variables (LVs) to use, computer generated cross-validation (venetian blinds) results were used. The risk of overfitting is minimized by choosing LVs that explain the maximum variance within the data at the minimum noise (root mean squared error; RMSE) based on the applied cross validation.

Discriminant compounds, which are compounds causing the separation/classification among the hop cultivars, are determined using a feature selection method called variable identification (VID) [11,12,13]. VID estimates the correlation coefficient between the volatiles and hop cultivars. Volatile with a VID coefficient value higher than absolute value of 0.70 were selected and identified.

Compounds were tentatively identified using the National Institute of Standards and Technology mass spectral library (NIST 14) with a match higher than 800 supported by comparing retention indices to literature values. Linear retention indices were calculated compared to elution of alkane series (C8-C20, Supelco, Bellefonte, PA, USA) injection in hexane. To further confirm the identity of these differentiating compounds, selected reference standards were injected using the same HS-SPME-GCMS method used for the hop samples. Compounds that were injected for further confirmation included; 1-octanol (BDH, Poole, England), γ-terpinene (Fluka, Buchs, Switzerland), β-caryophyllene (McCormick, Hunt Valley, MD, USA), D-limonene, methyl nonanoate, geranyl acetate and linalool (Sigma Aldrich, St Louis, MO, USA).

## 3. Results and Discussion

Nine commercially available and nine advanced selection hop cultivars were analyzed from the 2017 New Zealand harvest. The optimized HS-SPME-GC-MS fingerprinting approach enabled the detection of a wide range of volatile compounds across all of the cultivars. Data analysis was first conducted on the nine commercially available cultivars to identify differences between their volatile compound compositions (Section 3.1). This was then followed by a comparison of commercially available cultivars with advanced selection cultivars (Section 3.2).

### 3.1. Commercial Hop Cultivars

#### 3.1.1. Commercial Hop Cultivar Data Pre-Processing

**Figure 1:**
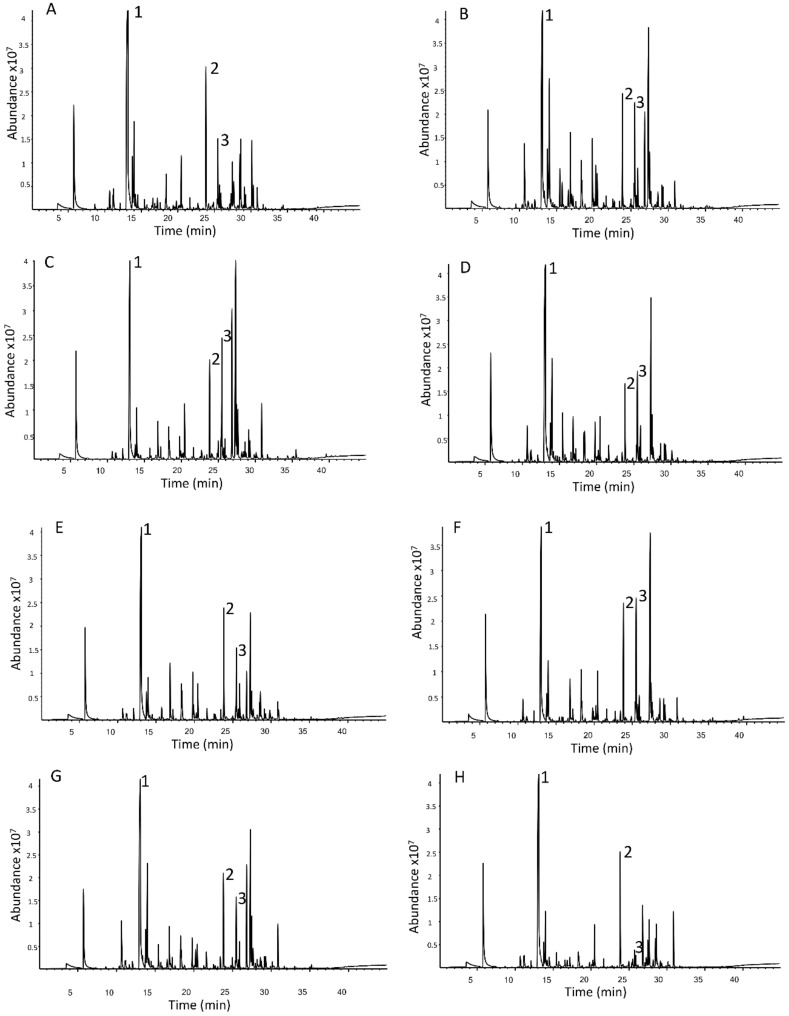

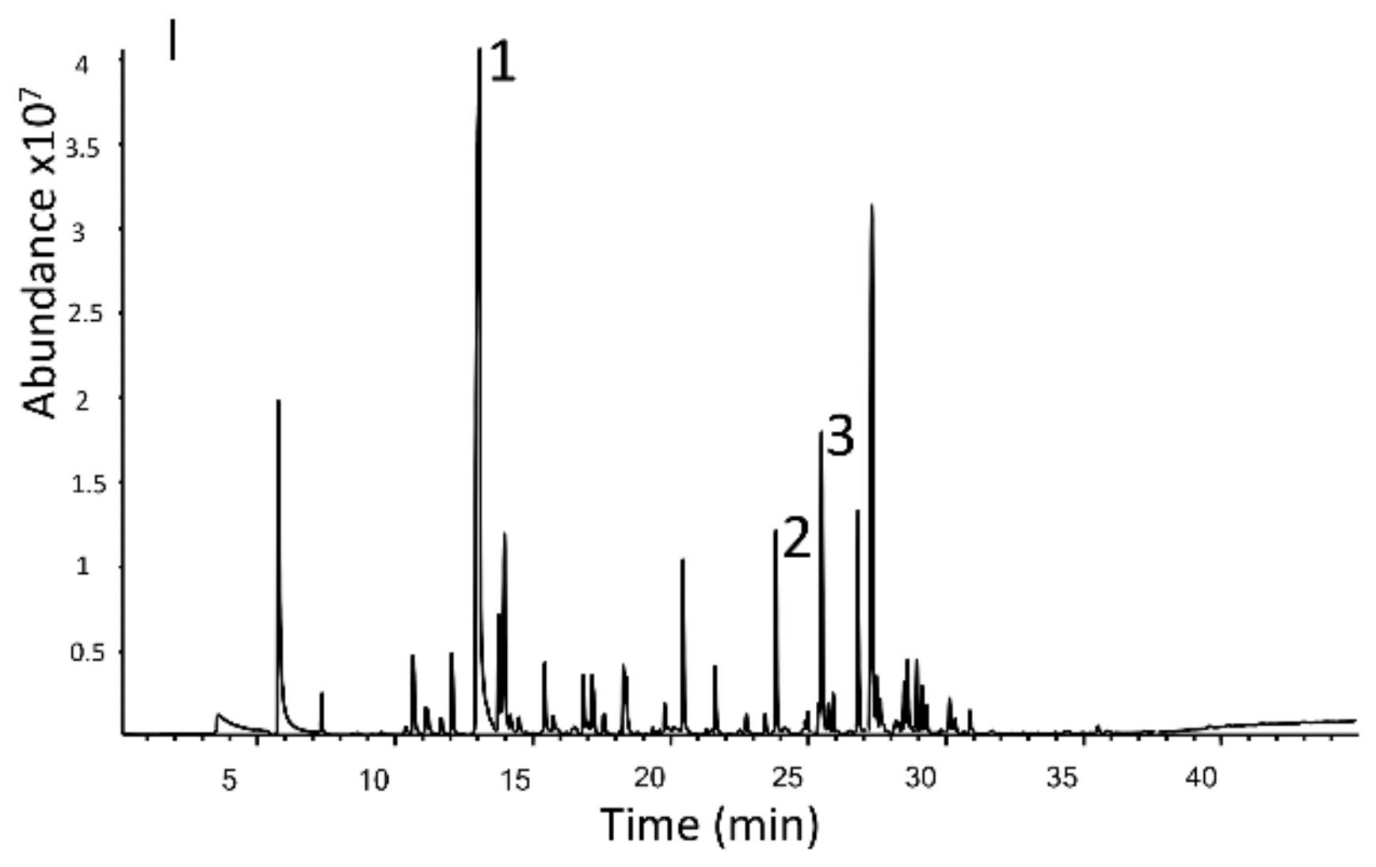
Total ion chromatogram of the volatile fraction of the nine hop cultivars obtained by the HS-SPME-GC-MS procedure. Chromatograms for cultivars; **A** = Riwaka, **B** = Rakau, **C** = Wai-iti, **D** = Nelson Sauvin, **E** = Wakatu, **F** = Kohatu, **G** = Waimea, **H** = Motueka, **I** = Taiheke. Compounds identified; 1 = β-myrcene, 2 = linalool, 3 = caryophyllene.

Over 120 volatile compounds, which consisted of many terpenoids, esters, alcohols, aldehydes and ketones, were detected across all hop cultivars. Figure 1 demonstrates a total ion chromatogram produced from each of the nine cultivars with three abundant compounds labeled. These exemplary chromatograms illustrate the range of volatile compounds that were detected using the HS-SPME-GC-MS fingerprinting procedure as well as the qualitative differences between volatile compounds across the cultivars.

Chromatograms illustrated that the method was reliable, volatiles were not overloaded on the SPME fibre and there were both qualitative and quantitative differences in peaks between cultivars. Although chromatograms appeared to have well defined peaks, co-elution of compounds did occur and deconvolution of these was achieved using AMDIS.

As the objective of the current study was to determine which volatile compounds are characteristic for different New Zealand hop cultivars, multivariate data analysis focused more on determining compounds that distinguish each cultivar rather than reporting the overall volatile composition. Thus, some of the more abundant peaks (e.g., β-myrcene and linalool) seen in all the chromatograms will not be reported, as these are present in relatively high amounts across all hop cultivars and thus do not discriminate.

#### 3.1.2. Comparing the Volatile Fraction of Commercial Cultivars Using Multivariate Data Analysis (MVDA)

Principal component analysis (PCA) and partial least squares discriminant analysis (PLS-DA) were used to analyze the multivariate data obtained with the headspace fingerprinting method. PCA was used as an initial unsupervised technique to explore if there were any outliers and to visualise groupings between the samples (Figure S1). Figure S1 shows a PCA bi-plot using the first two principal components. From the bi-plot, one outlier (Nelson Sauvin, subsample 1) was identified, which was then removed from the data set. Other than this outlier, reproducibility between sample replicates was reliable with only slight variations.

PLS-DA modelling was applied to describe the separation between the nine different commercial cultivars and select discriminant volatile compounds. For the PLS-DA model, detected volatile compounds were used as X-variables and the hop cultivars were considered as categorical Y-variables. Using venetian blinds cross-validation, 7 latent variables (LV) were selected for the model explaining 82.81% of the cumulative Y-variance (classification among the cultivars).

**Figure 2:**
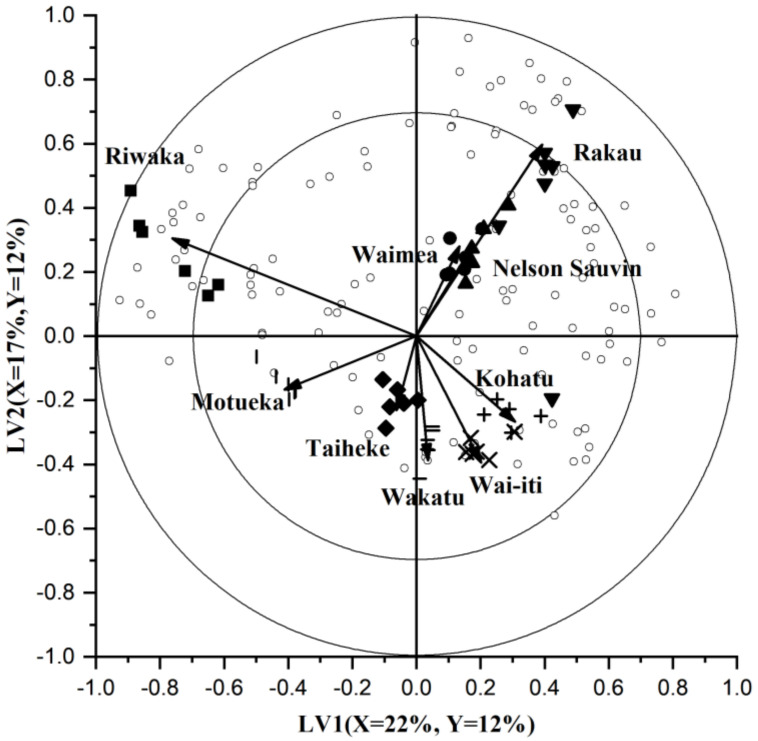
Partial least squares discriminant analysis (PLS-DA) bi-plot of nine commercial New Zealand hop cultivars using the first two latent variables (LV). X- and Y-variance explained by each LVs is also listed. Unfilled circles show the volatile compounds selected through gas chromatography mass spectrometry (GC-MS). Solid shapes represent the analytical replicates of each cultivar. Vectors represent contribution to the correlation coefficients for each cultivar. Rings represent correlation coefficients at 70% and 100%.

The PLS-DA bi-plot (Figure 2) is constructed using the first two highest LVs to show the similarities and differences between the nine commercial hop cultivars. The first two LVs of the PLS-DA model explain 39% of the X-variance (LV1 22%; LV2 17%) and 24% of the Y-variance (LV1 12%; LV2 12%). The low variance explained by the first 2 LVs indicate the high level of complexity within the data as a result of the number of cultivars examined. The PLS-DA projections using the higher LVs were also examined (LV2 vs. LV3 is illustrated in Figure S2), however LV1 and LV2 demonstrated the most variance and clearest understanding of how cultivars differed. The closer hop cultivars are to each other in the bi-plot, the more similarities they share in their volatile chemical profiles. The two rings on the bi-plot (Figure 2) represent the correlation coefficients at 70% and 100%. Each hop cultivar (Y-variable) has a representative vector, which represents the contribution to the correlation coefficients of LV1 and LV2. The unfilled circles represent the volatile compounds.

Figure 2 illustrates that Riwaka™ is projected far to the left of the biplot and has a long vector that expands past the first correlation coefficient ring. This demonstrates that it has a lot of difference in its volatile chemical composition compared to the other eight cultivars. Some of these compounds that separate the Riwaka™ cultivar from the others can be seen by the unfilled circles that are also projected to the left of the bi-plot and positively on the LV2 axis.

Rakau™ also has a large vector on Figure 2 that reached the 70% correlation coefficient ring. Surrounding this cultivar in the bi-plot are also lots of volatile compounds, which are likely to be present in higher amounts in the Rakau™ cultivar and is the reason why Rakau™ is projected away from the other cultivars. Figure 2 shows that the Rakau™ cultivar is positively projected on both LV1 and LV2. LV1 is what is separating Rakau™ from Riwaka™, however they have similar LV2 projections.

Nelson Sauvin™ and Waimea™ are projected in the same direction on the bi-plot (Figure 2) to Rakau™, however with a much shorter vector length. These two cultivars could be similar in their volatile compounds on LV1 and LV2, which could be further described by the volatile compounds surrounding them on the bi-plot. When examining the cultivars on other LVs, it was found that Nelson Sauvin™ and Waimea™ were separated from each other on LV3 (Figure S2).

Clusters of hop cultivars were seen in the lower right section of the bi-plot for Wakatu, Wai-iti™ and Kohatu^®^ cultivars. All three of these cultivars have a similar length vector and are projected relatively close to each other on the bi-plot, suggesting similarities in their volatile chemical compositions.

Further data analysis (feature selection) was conducted to classify what compounds were responsible for the discrimination between the nine hop cultivars (Section 3.1.3).

#### 3.1.3. Selection and Interpretation of Discriminant Volatile Compounds in Commercial Cultivars

A variable identification (VID) technique was used to select important volatile compounds that differentiate the hop cultivars from each other [19]. These values correspond to the correlation coefficient between each original *X*-variable and predicted *Y*-variable(s) by the PLS-DA model. Variables with an absolute VID value higher than 0.70 were considered important (discriminant compounds) and were identified using their mass spectra and retention indices. The discriminant components selected for each commercial cultivar are presented in Table 1. A positive VID coefficient indicates a higher detected amount of a component in the corresponding hop cultivar and vice versa for negative VID coefficients.

The VID technique is an effective method to determine volatile compounds that distinguish the cultivars from each other. Across all of the commercial hop cultivars, 61 differentiating volatile compounds were selected.

**Figure 3:**
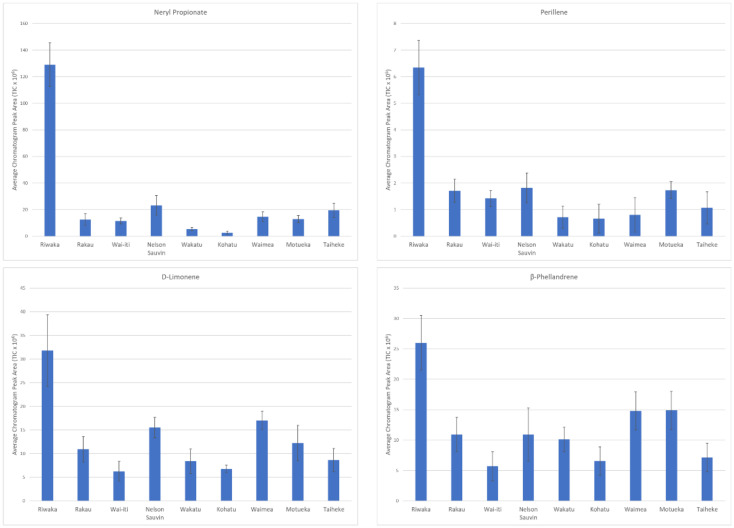
Bar graphs of four discriminant compounds found in the Riwaka cultivar showing the compound abundance in each of the hop cultivars measured (TIC × 10^6^). Error bars represent standard deviation.

Riwaka™ was separated from the other hop cultivars with the most distinct VOC profile, as illustrated in the PLS bi-plot (Figure 2). This separation was further confirmed by the 20 compounds found with a high VID, making it the commercial hop with the most differentiating compounds. Of these 20 compounds, 15 were only identified as discriminant compounds in Riwaka™ making these specific discriminant compounds for the cultivar. It was evident that the majority of the selected compounds for Riwaka™ were terpene derivatives (15 out of 20). The terpene hydrocarbon, β-phellandrene, was one of the differentiating terpene compounds, which had a VID of 0.83. This compound had been previously deemed as a discriminating odour compound in hops using GC-olfactometry [37]. The study listed that the compound had an odour intensity of 35.5% and was given odour descriptions such as ‘sulfury and catty’ [37]. Other differentiating compounds were limonene (VID = 0.86), perillene (0.94), neointermedeol (0.95), and two terpene esters, geranyl 2-methylbutyrate (0.96) and neryl propanoate (0.97). Figure 3 shows some of these differentiating compounds by concentration across all of the cultivars. The bar graphs illustrate that the compounds have the highest concentration in Riwaka™ cultivar and, therefore, that is why they were selected as differentiating compounds.

**Figure 4:**
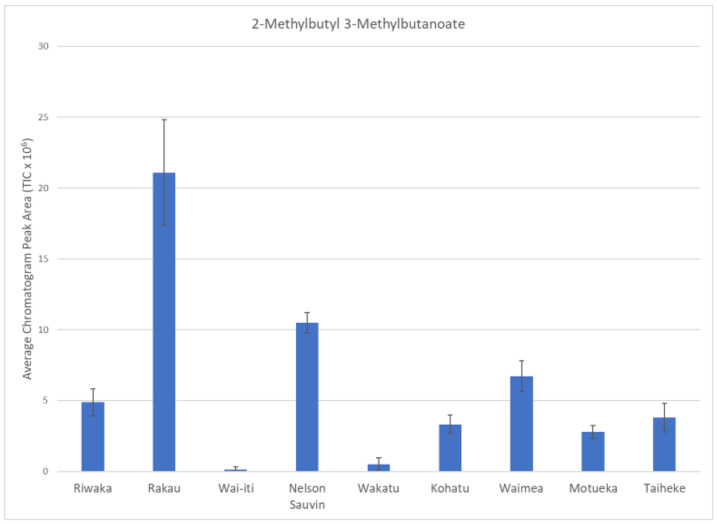
Bar graph of 2-methylbutyl 3-methylbutanoate showing the compound abundance in each of the hop cultivars measured (TIC × 10^6^). Error bars represent standard deviation.

Rakau™ had the second highest amount of differentiating compounds (14), many of which were branched chain esters, e.g., 2-methylbutyl 3-methylbutanoate (Figure 4). Branched chain esters have been previously reported as odour-active compounds in hops and beer [9]. These esters may be responsible for the fruity hop aromas that are present in the Rakau™ hop cultivar [38].

**Figure 5:**
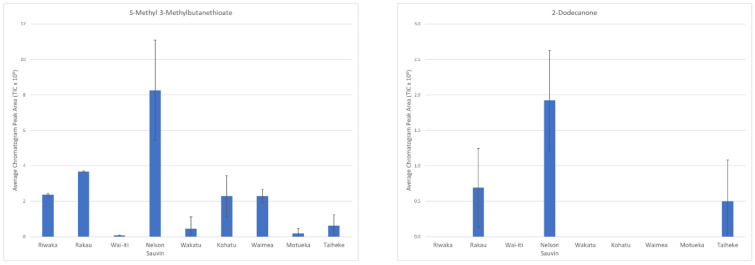
Bar graphs of discriminant compounds found in the Nelson Sauvin cultivar showing the compound abundance in each of the hop cultivars measured (TIC × 10^6^). Error bars represent standard deviation.

The top three compounds of the six that differentiated Nelson Sauvin™ from the other cultivars were s-methyl 3-methylbutanethioate (VID = 0.90), 2-dodecanone (VID = 0.86) and an unknown compound (VID = 0.84). Figure 5 shows the concentrations of s-methyl 3-methylbutanethioate and 2-dodecanone across the hop cultivars analysed. Nelson Sauvin™ is the most studied New Zealand hop cultivar due to its distinct white wine character that are different to other hop cultivars [17]. Polyfunctional thiols have often been focused on and a lot have been found in previous work studying Nelson Sauvin™ [9]. Although this study did not specifically target thiols due to the SPME method applied, the six differentiating compounds (Table 1) found in Nelson Sauvin™ add to the knowledge about what compounds distinguish it from other New Zealand cultivars.

In the visual representation of commercial hop cultivars (Figure 2), Waimea™ was very close to Nelson Sauvin™. When looking into the compounds selected (Table 1), Waimea™ only had two compounds that differentiated it from the other cultivars, namely 2-undecanol (0.91) and 2-undecanone (−0.72), which was negatively correlated. Although the two cultivars were very similar visually in Figure 2, they were discriminated on LV3 (Figure S2).

The clustering found between Kohatu^®^, Wakatu and Wai-iti™ (Figure 2) was suggested to be primarily due to aliphatic and terpene-related alcohol compounds, such as; 1-heptanol, β-eudesmol and cubenol. Taiheke^®^ was separated from this group by geranyl acetate (VID = 0.90). The clustering found between these cultivars is consistent with these cultivars sharing a common parent, Hallertauer Mittelfrüeh [6].

In general, the HS-SPME-GC-MS fingerprinting technique enabled the selection of a wide range of volatile compounds and identification of differentiating compounds in the commercial hop cultivars.

### 3.2. Comparison of Volatile Fractions of Commercial Cultivars and Advanced Selections

**Figure 6:**
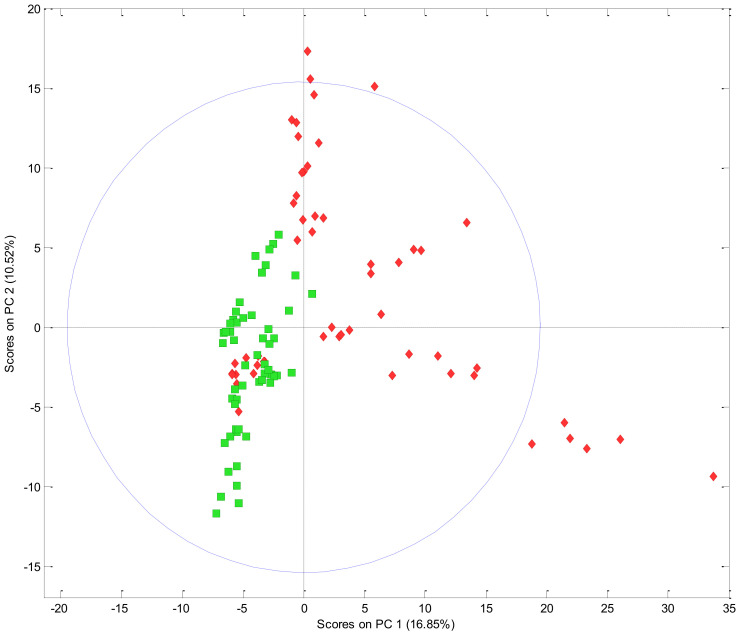
Principal component analysis (PCA) scores plot illustrating the difference between commercially available hop cultivars and advanced selections. Green squares = commercial hop cultivars, red diamonds = advanced selection hop cultivars. Points represent replicate analysis (*n* = 6). Individual hop cultivar samples are not identified on this figure due to confidentiality restrictions.

The integrated fingerprinting and chemometrics procedure was applied to investigate nine advanced hop selections (hop cultivars currently in the breeding programme), which were grown and harvested from the same location as the commercial hop cultivars. This data was combined with the commercially available hop cultivar data and analysed by PCA (Figure 6).

The PCA scores plot (Figure 6) shows a separation between the commercially available (green squares) and the advanced selection (red diamonds) hop cultivars based upon the volatile compounds detected in each hop cultivar. This separation suggests that some of the advanced selections possess unique volatile compound profiles, which may give rise to distinct flavours compared to NZ hop cultivars already on the market.

An overlap of two advanced selection cultivars and commercial cultivars can also be seen in the PCA scores plot, which illustrates a similarity in volatile chemical compounds between some of the cultivars. Similar patterns in volatile compounds could indicate where a new hop variety could substitute an existing commercial cultivar with a similar volatile profile. The overlap of advanced selection and commercial cultivars in Figure 6 relates to the parentage, which can be traced back to the Pacific Jade and Pacific Sunrise cultivars (Beatson, personal communication). The similarities of these cultivars can be further related back to their parentage cultivar, First Choice, which was a New Zealand hop that is no longer commercially available.

This information indicates that hop breeders in New Zealand are producing new hop cultivars that are different to the current commercial NZ cultivars in this study. Hop breeders can use this analytical approach to screen new cultivars in a targeted breeding scheme and the knowledge gained will guide the breeders to produce more hop cultivars with more distinct flavours in the future.

## 4. Conclusions

By using an untargeted chemical fingerprinting and chemometrics approach, this study was able to identify compounds in New Zealand hops that make them distinct from one another. PLS-DA highlighted similarities and differences between New Zealand commercial cultivars based upon the volatile compounds present in each cultivar. Riwaka™ was the most distinct cultivar with the highest number of compounds that differentiated it from the other hop cultivars, indicating this cultivar had more compounds that possessed the highest concentrations compared to the other hop cultivars in the study.

Similar volatile compound concentrations resulting in clustering of hop cultivars corresponded to hop cultivars with common parentage. This indicates the potential for further investigating genetic markers to understand the differences in the volatile compound concentrations as the phenotypic expression for each hop cultivar. This could further enable tools for targeted breeding of hop cultivars targeting specific compounds or chemical phenotypes.

Findings from this study should help New Zealand hop breeders understand more about how their hops (both commercial cultivars and advanced selections) differ from each other based on their volatile composition. The results illustrate the difference between commercially available and advanced selections, showing that the advanced selections are quantitatively different, which supports the aim of breeding distinct hop cultivars. New Zealand hop breeders in the future will be able to use this information to implement a systematic targeted breeding approach. This analytical approach could also be applied to improve the understanding of and facilitate targeted breeding of other crops to achieve specific phenotypic outcomes.

## Figures and Tables

**Table 1:** Discriminant volatile compounds, selected per hop cultivar based on the variable identification (VID) method.

		Retention Index **			
VID	Tentative Identification	Calculated	Reference		Chemical Class
**Riwaka**™					
0.97	Neryl Propanoate	1815	1831	[20]	Terpene Ester
0.96	Geranyl 2-methylbutyrate	1822	1819	NIST	Terpene Ester
0.95	*Unknown*	2081			
0.94	Perillene	1454	1431	[21]	Terpene
0.88	β-Eudesmene	1752	1717	NIST	Terpene
0.86	D-Limonene *	1211	1213	[20]	Terpene
0.86	*Unknown*	1542			Unknown
0.83	*Unknown*	1513			Unknown
0.83	β-Phellandrene	1223	1201	[22]	Terpene
0.79	α-Selinene	1757	1725	NIST	Terpene
0.79	Methyl 6-methyl heptanoate	1370	1338	NIST	Ester
0.79	cis-Chrysanthenol acetate	1648	1590	[23]	Ester
0.79	*Unknown*	1798			Unknown
0.77	Ipsdienol	1700	1678	[24]	Terpene Alcohol
0.75	cis-2-Menthenol	1600	1608	[25]	Terpene Alcohol
0.73	*Unknown*	1407			Unknown
0.73	Neointermedeol	2156	2138	NIST	Terpene Alcohol
0.72	γ-Terpinene *	1265	1246	NIST	Terpene
0.71	β-Pinene	1098	1097	[22]	Terpene
0.71	Caryophyllene oxide	1975	1989	NIST	Terpene Oxide
**Rakau**™					
0.93	2-Methylbutyl 3-methylbutanoate	1318	1299	[26]	Ester
0.93	S-Methyl hexanethioate	1440	1412	NIST	Ester
0.87	*Unknown*	1937			Unknown
0.84	cis-β-Ocimene	1270	1238	[27]	Terpene
0.84	Propyl 2-methylpropanoate	1035	1056	NIST	Ester
0.83	Methyl 4-decenoate	1674	1622	[28]	Ester
0.80	Methyl nonanoate *	1480	1487	[28]	Ester
0.79	2-Methylpropyl butanoate	1078	1094	[29]	Ester
0.76	2-Methylbutyl 2-methylbutanoate	1301	1285	[29]	Ester
0.74	Methyl decanoate	1627	1592	[28]	Ester
0.73	2-Methylbutyl 2-methylpropanoate	1204	1199	[29]	Ester
0.72	*Unknown*	1884			Unknown
0.71	*Unknown*	1289			Unknown
0.71	2-Methylpropyl 2-methylbutanoate	1180	1178	NIST	Ester
**Wai-iti**™					
0.91	Cubenol	2029	2084	[30]	Terpene Alcohol
0.86	Humulene-1,2-epoxide	2019	2071	NIST	Terpene Oxide
0.84	*Unknown*	2172			Unknown
0.78	2-Nonanol	1548	1508	[28]	Alcohol
0.76	β-Bisabolene	1748	1728	NIST	Terpene
0.75	1-Octanol *	1586	1561	[31]	Alcohol
**Nelson Sauvin**™					
0.90	S-Methyl 3-methylbutanethioate	1242	1225	NIST	Ester
0.86	2-Dodecanone	1686	1698	NIST	Ketone
0.84	*Unknown*	1232			Unknown
0.80	Methyl 5-methylhexanoate	1258	1247	NIST	Ester
0.76	*Unknown*	1795			
0.72	n-Propyl-2-methylpropanoate	1035	1054	[32]	Ester
**Wakatu**					
0.97	β-Eudesmol	2143	2188	[33]	Terpene Alcohol
0.93	2-Ethyl-1-hexanol	1519	1491	NIST	Alcohol
0.93	γ-Elemene	1672	1625	[34]	Terpene
0.91	Germacrene B	1846	1819	NIST	Terpene
0.89	α-Eudesmol	2138			Terpene Alcohol
**Kohatu^®^**					
0.86	Heptyl 2-methylpropanoate	1474	1433	[35]	Ester
0.85	*Unknown*	1276			Unknown
0.76	*Unknown*	1567			Unknown
0.72	1-Heptanol	1486	1453	NIST	Alcohol
0.71	1-Octanol *	1586	1561	[31]	Alcohol
**Waimea**™					
0.91	2-Undecanol	1732	1723	[36]	Alcohol
−0.72	2-Undecanone	1589	1598	NIST	Ketone
**Motueka**™					
−0.71	γ-Muurolene	1719	1681	[31]	Terpene
−0.77	Caryophyllene	1639	1595	NIST	Terpene
**Taiheke^®^**					
0.90	Geranyl acetate *	1770	1756	[31]	Terpene Ester

VID: Variable Identification, Retention Index, ** both retention indices from literature and calculated by injecting a series of C8-C20 alkanes injection, * Reference standard was used for identification confirmation. Compounds in ‘bold’ indicate those that were only identified as VIDs for the one cultivar, these can be looked at as potential characterizing compound. NIST: National Institute of Standards and Technology (https://webbook.nist.gov).

## Data Availability

The data presented in this study are available on request from the corresponding author. The data are not publicly available due to confidentiality.

## Supplementary Materials

The following are available online at https://www.mdpi.com/2304-8158/10/2/414/s1, Figure S1: Principal Component Analysis bi-plot of PC1 vs. PC2 for the 9 commercial hop cultivars, Figure S2, Partial Least Squares Discriminant Analysis showing latent variable 2 vs. latent variable 3.
