# Supplementary material for: Differences in New Zealand Hop Cultivars Based on Their Unique Volatile Compounds: An Integrated Fingerprinting and Chemometrics Approach
Source: Foods. 2021 Feb 13;10(2):414. doi: 10.3390/foods10020414 (PMC7917646; doi:10.3390/foods10020414)
Supplement: Supplementary file 1 [file foods-10-00414-s001.pdf]

foods

MDPI

Supplementary Material

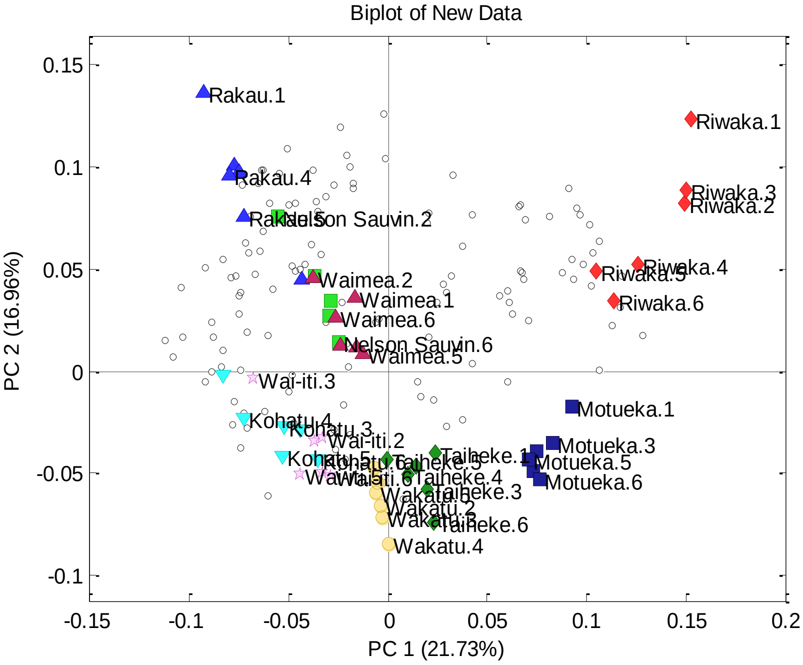

Figure S1. Principal Component Analysis bi-plot of PC1 vs PC2 for the 9 commercial hop cultivars.

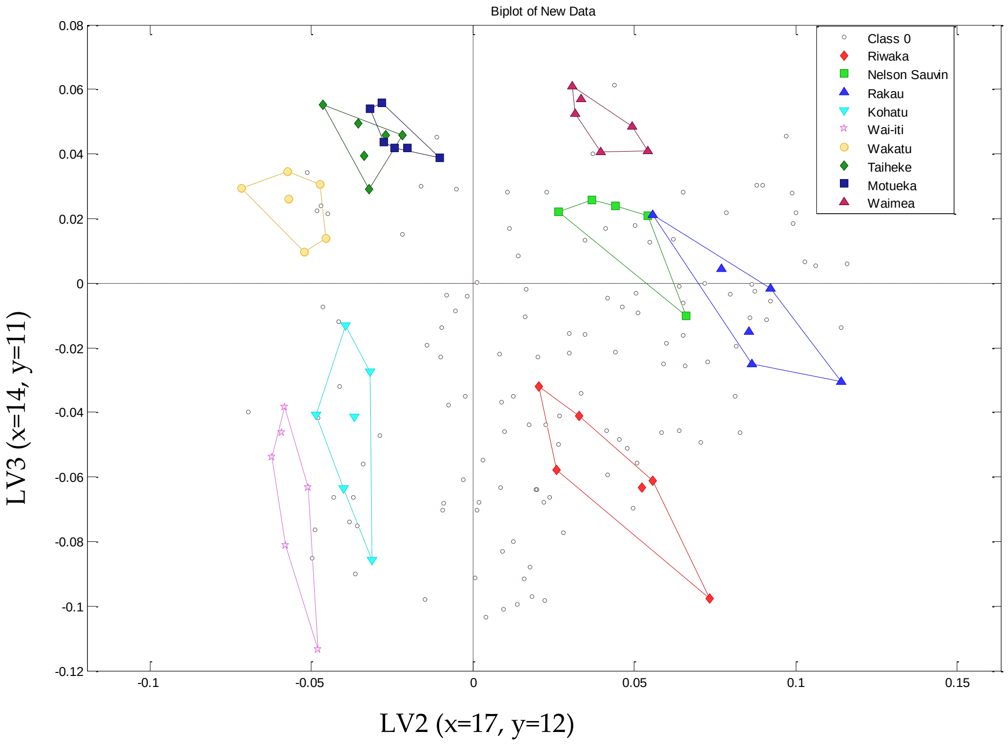

Figure S2. Partial Least Squares Discriminant analysis showing latent variable 2 vs latent variable 3. Different colours and shapes represent the 9 hop cultivars. Unfilled circles show the volatile compounds detected by HS-SPME-GCMS.
